# Immunity in Space: Prokaryote Adaptations and Immune Response in Microgravity

**DOI:** 10.3390/life11020112

**Published:** 2021-02-02

**Authors:** Macauley J. Green, Jonathan W. Aylott, Paul Williams, Amir M. Ghaemmaghami, Philip M. Williams

**Affiliations:** 1 School of Pharmacy, University of Nottingham, Nottingham NG7 2RD, UK; paxmg2@exmail.nottingham.ac.uk (M.J.G.); jon.aylott@nottingham.ac.uk (J.W.A.); 2 School of Life Sciences, University of Nottingham, Nottingham NG7 2RD, UK; paul.williams@nottingham.ac.uk (P.W.); amir.ghaemmaghami@nottingham.ac.uk (A.M.G.)

**Keywords:** microgravity, spaceflight, immunology, pathogens, macrophages, bacteria, viruses, innate immune response, adaptive immune response

## Abstract

Immune dysfunction has long been reported by medical professionals regarding astronauts suffering from opportunistic infections both during their time in space and a short period afterwards once back on Earth. Various species of prokaryotes onboard these space missions or cultured in a microgravity analogue exhibit increased virulence, enhanced formation of biofilms, and in some cases develop specific resistance for specific antibiotics. This poses a substantial health hazard to the astronauts confined in constant proximity to any present bacterial pathogens on long space missions with a finite number of resources including antibiotics. Furthermore, some bacteria cultured in microgravity develop phenotypes not seen in Earth gravity conditions, providing novel insights into bacterial evolution and avenues for research. Immune dysfunction caused by exposure to microgravity may increase the chance of bacterial infection. Immune cell stimulation, toll-like receptors and pathogen-associated molecular patterns can all be altered in microgravity and affect immunological crosstalk and response. Production of interleukins and other cytokines can also be altered leading to immune dysfunction when responding to bacterial infection. Stem cell differentiation and immune cell activation and proliferation can also be impaired and altered by the microgravity environment once more adding to immune dysfunction in microgravity. This review elaborates on and contextualises these findings relating to how bacteria can adapt to microgravity and how the immune system subsequently responds to infection.

## 1. Introduction

The immune system is influenced by external stressors and adapts accordingly. The differential immune response under microgravity gives rise to complex immunological issues [1,2] which will be discussed in this review. For instance, 15 of the 29 Apollo mission astronauts incurred viral or bacterial infections during their mission or within a week upon returning to Earth [3]. Many bacteria grown under microgravity experience physiological changes, increased virulence, and differential antibiotic susceptibility amongst other changes [4,5,6]. Such changes can work synergistically to cause an increased chance of infection with a chance of enhancing the potential for a poor prognosis. However, the microgravity environment may also provide novel insights into different biological phenomena here on Earth such as T cell exhaustion [7] and T cell ageing [8]. 

The last century has seen the frontier of space become accessible by human beings. From Yuri Gagarin’s initial flight into space, through NASA’s (National Aeronautics and Space Administration) moon landing in 1969, progressing to ventures in space tourism and planned manned missions to Mars in 2030, space has become an environment that is habitable for periods of time by humans. As missions to space become longer and further away in distance, the time spent in the space environment increases and with it, the associated health risks [9,10]. One major differential factor from the environment on Earth is the change in gravity to a near-zero state known as microgravity.

Most research on the effects of microgravity on biology either aims to help provide insight on how to keep astronauts healthy on prolonged missions into space or to use microgravity as a research tool to further understand the biology to be beneficial to patients on Earth. The focus of this paper is to summarise the impact of microgravity on the immune response to bacterial infection and the individual changes to bacteria and immune cells.

## 2. Microgravity Simulation and Applications

Microgravity is the condition when objects appear to be weightless. Studies have been conducted on true microgravity in space since the initial Apollo missions and in microgravity analogues as early as the 1980s. True microgravity has been regularly investigated onboard SpaceLab, the Russian Mir space station, and the International Space Station (ISS). As well as experiencing true microgravity, onboard samples and experiments are subjected to time dislocation, elevated carbon dioxide levels and low dose cosmic radiation [11]. Some of these factors can be synergistic, for instance, radiation induces oxidative stress in the skeletal system and microgravity increases the oxidative stress-induced [12]. However, resources are limited on these stations and sending samples and consumables for experiments to these platforms is expensive and requires substantial time to plan and execute. A solution to this problem is to create an environment analogous to the low-shear environment created by microgravity on Earth. This has the benefit of being more time and cost-effective and the microgravity variable can be separated from other space variables more easily. These microgravity analogues still are subjected to 1 g but create a low-shear environment through various means as discussed in the following sections. In true microgravity, the overall net force of gravity is in the range of 10^−6^ compared to the force of gravity at the Earth’s surface.

### 2.1. Microgravity Analogues—The Common Devices

**Figure 1:**
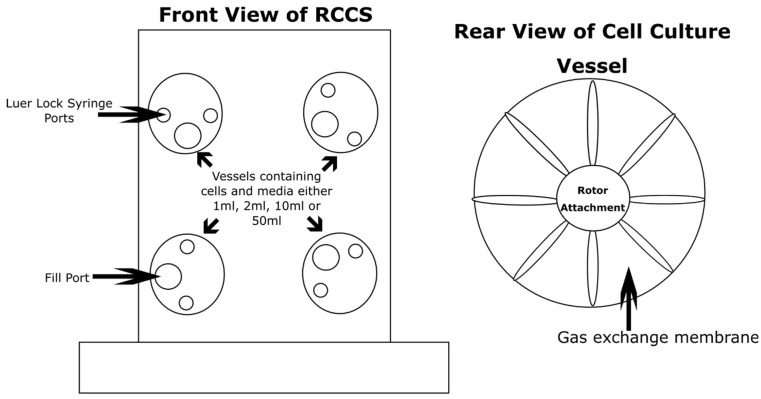
The 2D clinostat; this one is known as the Rotating Cell Culture System (RCCS). The particular version illustrated in this figure is capable of rotating up to four individual cell culture vessels. These cell culture vessels carry media containing cells in 1 mL, 2 mL, 10 mL, or 50 mL formats. The cell culture vessels come in two varieties, disposable and autoclavable. The vessels compose of two Luer Lock syringe ports for small additions or extractions to the growth medium and a larger fill port for ease of filling and emptying. The rear of the vessels composes of a gas exchange membrane to allow the diffusion of gases. The vessels rotate clockwise at independent or synchronous speeds.

Microgravity creates a low-shear culture environment since convection currents are absent. By creating a low-shear environment on Earth, low-shear responses of biological samples can be investigated and used to theorise responses in other low-shear environments such as microgravity. One method of creating a low-shear environment is the rotating cell culture system (RCCS) (Figure 1). This bioreactor was designed by NASA and is commonly used across Europe and the USA via production and distribution from Synthecon [13,14]. This bioreactor employs solid body rotation around a horizontal axis to minimise fluid shear forces on the sample whilst keeping it in suspension. This is achieved through rotation at a precise speed where this phenomenon occurs. Shear force is the application of a force perpendicular to a surface. The difference in velocity between the layers in moving liquid result in shear forces being imparted on the liquid and samples contained within. By rotating the liquid containing vessel at a precise speed, shear forces can be minimised, hence ‘low-shear’. This needs to be fine-tuned with respect to the weight of the sample to prevent sedimentation and keep the samples in ‘freefall’ by balancing the net shear force with gravity on the sample. This creates an analogous environment in the low-shear environment created by true microgravity.

**Figure 2:**
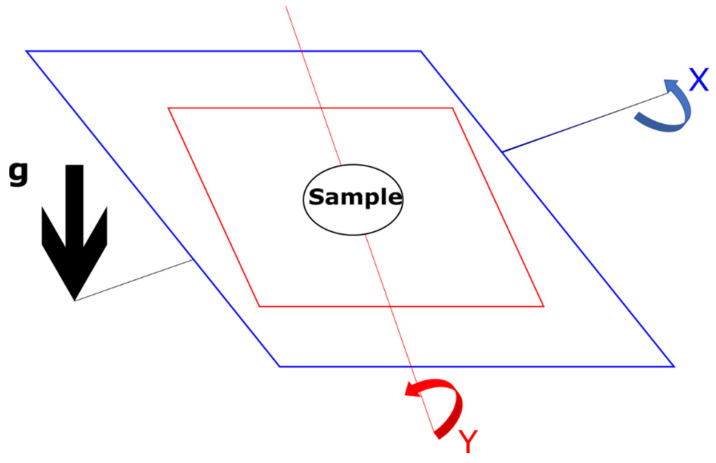
A schematic of how the Random Positioning Machine (RPM) simulates microgravity. This schematic is based upon the illustration from Wuest et al. 2017 [16]. The sample in the centre of the device is constantly repositioned both in the direction of the *x*-axis and the *y*-axis, giving an overall net-zero gravity vector.

The random positioning machine (RPM) (Figure 2) is another common tool for creating a low-shear environment for the biological samples by creating shear forces away from the centre where the sample is. Utilizing constant repositioning upon a dual axis, this instrument causes the overall net influence of gravity to be zeroed at long time scales [15]. Both the RWV and RPM are two examples of a 2D clinostat.

### 2.2. Microgravity Analogues—History of Clinostats and Alternative Methods

Clinostats rotate samples around one or more axes and were developed in the late 1800s when gravity was discovered to be a major factor in plant growth [17]. This goes back to as early as 1806 and the use of a water wheel to generate altered gravity environments [17]. Slow rotation around an axis (1–2 rpm) was found in the 1980s to induce ultrastructural disturbances not found in the microgravity environment [18]. The first study to use a faster rotation with a clinostat was Briegleb in 1992 [19]. 

Diamagnetic levitation is another microgravity analogue. This is the use of a high gradient magnetic field that can levitate a biological sample [20]. This method, however, does not negate phenomena not observed in microgravity, such as convective stirring of liquid that increases oxygen availability in the samples, which is a major issue in space as convection is not present [20,21].

Parabolic flight and drop towers are also employed by researchers for short microgravity exposure. During parabolic flight, an aeroplane will fly in parabolic arcs imparting approximately 30 s of free-fall-per-parabola. The nature of the flight path causes the imparted gravitational forces to fluctuate between microgravity during freefall and 1.8 g during the reversal of the flight path at the bottom of the parabolic flight path. These short fluctuating periods of microgravity and hyper-gravity have already been used in immunological research [22,23]. Drop tower samples are dropped from a substantial height in a capsule covered by either an aerodynamic drag shield or in a vacuum to allow free-fall at 9.8 m s^−2^. This enables the samples to experience near weightlessness as they freefall from a substantial height. NASA, for example, use a 24.1-metre drop tower that enables 2.2 s of microgravity to be experienced.

Microgravity analogues, however, are not an entirely accurate model of orbital microgravity conditions. Gene expression analysis of human renal cortical cells cultured during spaceflight and in an RCCS on Earth show that 700 more genes with a total of 1600 had altered expression levels compared to 900 genes with altered expression in the RCCS [24].

## 3. Prokaryotic Responses to Microgravity

Prokaryotes have evolved and adapted to survive in a plethora of different environmental conditions [25]. Microgravity is a different environmental condition that, due to advances in technology, is becoming a condition for prokaryotes to adapt to and can be researched and explored. This area of research could be beneficial for combatting infection during long term manned space missions and may provide novel insights into prokaryote adaptability and evolution. The following sections review the research into the prokaryotic response to the low-shear environment created by microgravity and ground-based analogues.

### 3.1. Cell Viability and Diversity

The human body itself contains a substantial number of bacteria from the bacteria covering the skin to the microflora of the gut [26,27]. NASA has set acceptability limits for bacterial numbers in the air, on surfaces and in water for all space-bound equipment and vessels.

As shown in Table 1, it is expected that bacteria during spaceflight will survive and proliferate in microgravity/spaceflight conditions. This is shown by the higher bacterial acceptability limits for air and surfaces inflight compared to preflight.

Vessels from different locations unsurprisingly show differences in bacterial populations. The Russian Mir space station reported the most dominant genera of airborne and surface bacteria to be *Staphylococcus* with *Sphingomonas* and *Methylobacterium* to be the most dominant genera in the potable water [29]. When water was collected from the ISS between 2009–2012, it was found the most common organisms were *Burkholderia multivorans* and *Ralstonia pickettii* with air and surface dominance of *Bacillus*, *Micrococcus,* and *Staphylococcus* species [28]. Overall, the most common phylum was Actinobacteria [30].

### 3.2. Overview of Previous Studies

The following studies have been undertaken to investigate the effects of a low-shear environment on bacteria with a few studies also investigating archaea. The first studies found common responses to spaceflight bacteria with emphasis on the phenotypic responses including but not limited to: changes in growth rate, resistance to external stresses and varying effects on bacterial conjugation [14]. Below is a brief summary of the major studies investigating individual species and strains of bacteria and their critical findings.

### 3.3. Transcriptomic Changes

To summarise the transcriptomic results from the studies in Table 2, common dysregulated genes have been identified and hypothesised as being altered because of microgravity.

The global post-transcriptional regulator Hfq is one of the genes that has been identified to show altered levels of expression across multiple pathogenic species of bacteria in both microgravity and microgravity analogues [43,45]. This gene is found approximately in half of all known bacterial genomes and plays an important role in bacterial stress responses [45]. Hfq is an RNA-binding chaperone protein whose activity regulates bacterial protein expression via small bacterial RNAs (sRNAs) [47]. The latter regulate many bacterial processes and have a length usually ranging between 50–500 nucleotides [48]. They act via antisense mechanisms on multiple target mRNAs and exert global effects on factors such as virulence, stress responses and adaptive metabolic changes [49].

The ferric uptake regulator (Fur) and its homologues; the zinc uptake regulator (Zur), the manganese uptake regulator (Mur), and peroxide stress defence control regulator (PerR) [50] are required in some microgravity analogue stress responses in e.g., *Escherichia coli* [51]. Fur is a transcription factor which represses siderophore synthesis in pathogens by utilising Fe^2+^ as a corepressor [50]. Many low-shear environment response genes are found in clusters or operons [36] and upstream of many of these operons is a Fur binding site. Regulation of the low-shear response via Fur has been shown with a *Salmonella fur* mutant which is consistent with Fur transmitting the microgravity analogue signal [36]. For the acid resistance response to microgravity analogue regulon, *fur* is found upstream. When exposed to a low-shear environment, the *Salmonella* strain used in the study showed increased acid resistance whereas the Fur mutant strain showed no increase in acid resistance [36]. This strengthens the hypothesis of the Fur protein regulating a microgravity stress response regulon; however, more studies are needed.

General stress responses in *E. coli* and many other bacteria are regulated by the sigma subunit of RNA polymerase known as RpoS [52,53]. 

Interestingly, this is not the case for microgravity analogue response in a rotating cell culture system in *Salmonella enterica serovar Typhimurium* which adapts in an RpoS-independent manner to environmental stresses [32].

### 3.4. Antibiotic Resistance

A major finding of note for bacteria grown under microgravity is the increase and differences in biofilm formation, architecture, and the development of antibiotic tolerance.

Antibiotic resistance poses a severe health risk both in spaceflight and once the astronauts return to Earth. Upon return to Earth, an antibiotic-resistant strain may spread through the population. Furthermore, microgravity is just one factor during spaceflight that has been shown to increase antibiotic resistance [54]. The bacterial adaptive response, which is the exposure to a sub-lethal stressor which induces resistance to a lethal level of the same or different stressor [55], can also be triggered by ionising [56] and non-ionising radiation [57] found as part of the cosmic radiation [54]. Radiation may cause changes to antibiotic efflux pumps and sensitivity to chemicals [56]. Antibiotic resistance profiles (see later) and biofilm formation are not generic responses to extreme environments. A comparative study of *Staphylococcus* and *Enterococcus* isolates from the ISS and the Antarctic Research Station Concordia were compared and the ISS isolates were found to be more resistant to the antibiotics tested [58]. This could indicate non-space extreme environment studies may not be good substitutes or generate comparable data to the study of the extreme environment of space. However, more comparisons are needed. This could also mean microgravity and/or other space stressors may trigger the expression of different genes in the response to extreme environments.

Long term microgravity analogue studies have been performed to simulate long term manned missions to try and predict antibiotic resistances that could potentially evolve. One such study used the RCCS for 1000 generations of *E. coli* over which it became tolerant to cefuroxime, chloramphenicol, cefalotin, cefuroxime axetil, tetracycline and cefoxitin [59]. Interestingly, after a further 110 generations in Earth gravity conditions, chloramphenicol and cefalotin resistance was retained. This could be due to an accumulation of mutations.

During spaceflight, and especially on a long-distance manned mission to Mars, there will be a finite amount and diversity of medications. Especially with the longer manned missions, there is no feasible way to restock the vessels or send new medications/antibiotics. Therefore, if a multidrug-resistant strain develops and becomes resistant to the antibiotics on board the vessel then all the passengers’ lives are at risk as the infection may not be treatable. 

Additionally, this can also pose a threat to health on Earth. In microgravity, the bacteria may develop antimicrobial resistances that are different from those that develop in Earth’s gravity. This could enable widespread infection and disease on return to earth if the pathogenic bacteria have infected one of the passengers and this has gone unnoticed.

Virulence is defined as the ability of the bacteria to cause disease and can also be referred to as pathogenic potential [60]. Increases in virulence have been reported in both analogue and spaceflight microgravity [6,61,62]. With regards to spaceflight, this is especially of concern due to the constant close contact with other astronauts in the relatively small space vessel. Increased virulence combined with antibiotic resistance poses a massive health risk and will greatly increase the dangers of both acute and chronic infections.

### 3.5. Archaeal Responses to Microgravity

Archaea are distinct from bacteria and are prevalent in extreme environments and are also a natural component of the microbiota of humans [63]. However, no known pathogenic archaea exist [63]. Table 3 summarises studies of archaea in a low-shear environment.

Haloarchaea are the most studied area species that live/survive in aqueous environments i.e., water. Some haloarchaea show an increase in antibiotic resistance which may be a problem as archaea and bacteria can undergo horizontal gene transfer, especially from archaea to bacteria [66,67]. Horizontal gene transfer is the acquisition of new genetic material from another organism, this is a major driver of bacterial pathogen evolution and antibiotic resistance [68].

The consequences of infection are not solely dependent on the pathogen trying to infect the host. The immune response is vital in clearing infection and conferring future immunity. Microgravity has a severe impact on the immune system both as a whole and on its individual components and will be discussed in the following sections. Additionally, microgravity and other space stressors such as radiation, sleep deprivation, isolation and microbial contamination have been shown to suppress immune function [54]. This review will summarise how the natural defence against pathogens is affected and how the crosstalk between immune cells and bacterial pathogens is also altered by the microgravity stressor. 

## 4. Immune Cell Responses to Microgravity

The immune system is composed of two different major systems, the innate immune system and the adaptive immune system. The innate response is commonly referred to as the non-specific response and usually occurs immediately or within hours after the appearance of an instigating antigen [69]. The innate immune system consists of physical barriers such as the skin and mucus and cells such as monocytes, macrophages, neutrophils, natural killer cells, mast cells, basophils and dendritic cells [70]. The adaptive immune system is commonly referred to as the acquired immune system and occurs at a later time point than the innate immune system. The adaptive immune system consists of lymphocytes known as T-cells and B-cells which specifically target the pathogen and provide future protection against that pathogen [71]. The following sections of this review will outline how both systems are affected by a low-shear environment and how this impacts on their response to bacterial infection.

**Figure 3:**
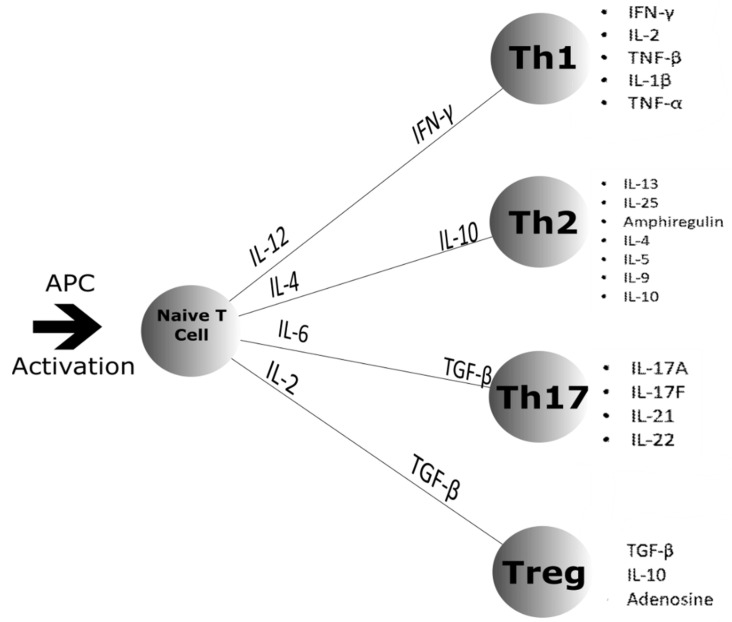
Naïve T cell differentiation after Antigen Presenting Cell (APC) activation. The naïve T cell differentiates into four different classes of T cell: Th1, Th2, Th17 and the Treg. APC activation causes the release of the cytokines shown along the lines that signal for which class the naïve T cell will differentiate into. Once differentiated, the T cells secrete the cytokines shown by the bullet points to the right [79,80,81].

Major changes in immune system function occur during spaceflight [72]. Circulating monocytes, T-cells, B-cells and neutrophils are all increased with a decrease in natural killer cells [73]. Distribution of peripheral leukocytes is altered with specific subpopulations showing diminished function [1]. Latent viruses such as herpes reactivate [74,75] and indicate compromised adaptive immune function [1]. Epstein-Barr virus, cytomegalovirus and VZV (human neurotrophic alpha herpes virus) have also been reported to be reactivated during spaceflight [73]. Hypoplasia of the spleen can also occur with an increase in peripheral blood neutrophils [76,77]. Natural killer cells exhibit lower cell cytotoxicity and there is also a delayed response to hypersensitivity skin tests [76]. One reason for the inhibition of natural killer cell toxicity is reduced production of granzyme B and perforin with effects being reported up to 60 days after spaceflight [78]. B cell activation in microgravity is still largely unknown but short-term flights have shown no significant changes [76]. This is an interesting finding as the same study suggests a Th2 shift occurs in microgravity which may affect immunoglobulin production. Figure 3 shows the normal differentiation pathways for naïve T cells. However, this is only a hypothesis and due to a decrease in Th1 cytokines being present [76]. 

Studies of long-term space missions on B cell activation and immunoglobulin production is thus far inconclusive [82,83,84,85].

Due to the nature of the low-shear environment, motility of immune cells is greatly reduced [76]. This combined with reduced monocyte motility and cytoskeletal modifications may lead to the reduced interactions between monocytes and lymphocytes which has been shown to be essential for costimulatory signalling [76].

### 4.1. Cell Differentiation

Differentiation inhibition has been reported by a plethora of immunological studies in a low-shear environment created by both microgravity and ground-based analogues [15,86,87,88,89,90,91].

The reasons for differentiation inhibition have been greatly speculated upon. One school of thought is that non-differentiated monocytes are suspension cells that become adherent upon differentiation. Future investigations into whether the low-shear environment prevents adherence and therefore the differentiation of the cells could warrant interesting results.

More recent studies [89] have delved into altered pathways due to low-shear forces to shed light on the differentiation inhibition problem. RAS/ERK/NF-κB pathway was shown to be a low-shear regulated pathway, where exogenous ERK and NF-κB activators were able to counteract the effects of microgravity on macrophage differentiation in both microgravity and ground-based analogues [89]. This study also verified via qPCR and western blot that the p53 pathway was also affected by the low-shear environment. This concurs with older studies which also conclude that altered genetic pathways cause immune cell differentiation inhibition [92,93]. Furthermore, cell cycle ‘arrest and progression’ proteins have been shown to be altered. P21 increases 4.1-fold in 20 s of spaceflight microgravity culture in primary cells and 2.9 times in Jurkat T-cells compared to ground controls. These results suggest that cell cycle progression is gravity dependent in T-cells and can halt the progression of differentiation [94]. Additionally, these results were confirmed by other studies [95].

Differentiation into effector T-cells is also driven via dendritic cells through the production of IL-2. The alterations in IL-2 production that mimic T cell exhaustion also provides an explanation for T cell resistance to differentiation into effector T cells [96].

The surrounding microenvironment provided by the connective tissues can also have immune-regulatory effects. Mesenchymal stem cells (MSCs) are stromal cells that can differentiate into connective tissues and are integral to some specific immune responses. They do this via the production of cytokines and molecules such as but not limited to; PGE2, nitric oxide, FasL, PD-L1/2, IDO and IL-6 [97]. Culture in a low-shear environment maintains the undifferentiated state of MSCs [91] as mechanical loading is an important determining factor for osteogenic differentiation [98]. This may potentially be due to the downregulation of the master osteogenic transcription factor Runx2 and main osteogenic differentiation markers ALPL and OMD in long term microgravity analogue culture [87]. Low-shear culture also affects myogenic differentiation [98]. During spaceflight, 1599 genes have altered expression with important changes being a reduced expression of cell-cycle genes which leads to cell proliferation inhibition [99].

### 4.2. Pathogen Recognition

A few co-culture studies have been conducted to investigate how immune cells and bacteria respond to each other in a low-shear environment. Macrophages co-cultured with *S. enterica serovar Typhimurium* showed activation of the stress associated mitogen-activated protein kinase, kinase 4 in a ground-based analogue [100]. Furthermore, the bacteria themselves had an augmented invasive potential and increased tumour necrosis factor-alpha (TNFα) production in infected epithelial cells [100]. The same study also found increased production of *E. coli* heat-labile enterotoxin in co-cultures [100]. Finally, it was also shown by this study that murine macrophages infected with enteropathogenic *E. coli* also showed increased production of TNFα [100]. Furthermore, co-cultures have also shown that in a low-shear environment, monocytes have reduced ability to engulf *E. coli* [76]. CD32 and CD64 which are involved in phagocytosis have also been shown to be reduced in surface expression [101].

Lipopolysaccharides (LPS) are major membrane surface components that are endotoxins present in most Gram-negative bacteria with a few rare exceptions and are strong stimulators of innate immunity [102]. Stimulating immune cells grown in a low-shear environment with LPS therefore provides insight into how immunological crosstalk occurs during spaceflight and the response of the immune system to endotoxins. A major biochemical response after the challenge with LPS is p38 MAP kinase activation via phosphorylation [103]. P38 mitogen-activated protein (MAP) kinases are one of four main sub-groups of MAPs that mediate cellular behaviours in response to external stimuli [104], which potentially includes microgravity. It was hypothesised that p38 MAP kinase would be sensitive to microgravity as there are various genes such as PRKCA that are regulated by p38 MAP kinase and are sensitive to a low-shear environment [105]. However, monocytes exposed to spaceflight did not show impairment of p38 MAP kinase and actually showed a slight increase in activation [106]. *Caenorhabditis elegans* in analogue culture increased transcriptional expression of three genes that encode the core p38 MAPK pathway and expression of phosphorylated PMK-1/p38 MAPK [107]. These genes were pmk-1, nsy-1 and sek-1 [107].

Stimulating immune cells with LPS also causes NF-κB (nuclear factor-kappa B) to translocate from the cell cytoplasm to the nucleus. This translocation is altered in many cell types in microgravity and analogues [108]. A study on human Jurkat T cells showed decreased translocation of NF-κB in parabolic flight and ground-based analogues, [109] and two studies on activated human T cells via RT-qPCR and microarray on whole cell lysates [110,111] showed suppressed expression of NF-κB gene targets. An interesting connection can be made between 1g and low-shear environment studies to theorise why the translocation is altered. NF-κB has been shown to be MyD88 dependent [112] and furthermore, potential immune blunting of cells due to the low-shear environment causes suppression of MyD88 [113]. MyD88 encodes for proteins involved in the early uptake of LPS [113]. This may explain why some studies show inhibition of NF-κB translocation in a low-shear environment from both microgravity and ground-based bioreactors.

LPS stimulation also elicits ROS (reactive oxygen species) production in macrophages. This was investigated in a microgravity analogue via Syk phosphorylation [114]. Syk phosphorylation was significantly reduced in microgravity when macrophages were stimulated by LPS, zymosan or curdlan [114], revealing that ROS production in macrophages is sensitive to gravitational forces. Other studies confirm this by showing ROS production in various cell types increases in a microgravity analogue [115]. The study also found that NF-κB signalling was unaffected by the microgravity analogue which is a later step in the signalling cascade than Syk phosphorylation, and inconsistent with the studies previously discussed. This work resulted in the proposal of a hypothesis that during long spaceflights the immune system may be able to adapt to microgravity effects [114]. Additionally, in macrophages in a microgravity analogue, TNFα but not IL-1β was suppressed following stimulation with LPS [116].

LPS is not the only bacteria-derived stimulus of the immunological response to bacterial infections. This is due to recent findings suggesting that LPS stimulation may not be affected by a low-shear environment [117]. LPS and pokeweed mitogen stimulation both failed to alter levels of TNFα and IL-10 release in whole blood [117]. The overall findings of this study concluded that the IL-2 and interferon-gamma responses to immune cell mitogen and antigen stimulation are inhibited by a microgravity analogue whereas TNFα and IL-10 secretion are greatly influenced by a microgravity analogue [117]. These results also corroborate the spaceflight sample results [2]. Mitogen stimulated immune cells showed reduced production of interferon-gamma, IL-10 and TNFα just like the microgravity analogue results. This study additionally showed reduced production of IL-6 and IL-5 [2]. A major contrast between the findings was that in a microgravity analogue via a random positioning machine, LPS stimulation did not alter levels of IL-10 production compared to ground controls whereas during spaceflight IL-10 production was reduced during LPS stimulation. These variations may be due to differences between microgravity analogues and true microgravity or it could be as a result of the differing conditions of space other than microgravity. IL-8 production was also increased during LPS stimulation in spaceflight which is concurrent with other studies [113]. Transcriptomic analysis of the immune cells during spaceflight shows suppression of MyD88, MD-2, and Lbp which are responsible for encoding [113] proteins that are involved in the early uptake of LPS [113].

The fascinating area of interest arising from comparing these studies is the difference in interleukin expression upon LPS stimulation depending on whether cells were cultured in a microgravity analogue or true microgravity. Transcriptomic analysis of LPS stimulated immune cells grown in a microgravity analogue compared to the spaceflight analysis of Chakraborty et al. 2014 may be able to add clarity to these different results. 

### 4.3. Cell–Cell Interactions

Cell–cell interactions are severely impacted by microgravity and low-shear analogues. Dendritic cells play a vital role in recognising pathogens and activating T-cells. Murine dendritic cells (JAWSII) have recently been cultured in the rotary cell culture system for 2–14 days to determine the impact of microgravitational changes both short term (less than 72 h) and long term (4–14 days) [118]. Short term culture was shown to enhance the T-cell activation of dendritic cells through increased expression of surface proteins that are associated with maturation and interleukin-6 (IL-6) production [118]. Other dendritic cell studies in the rotary cell culture system have shown that T-cell resistance to activation in a microgravity analogue mimics T cell exhaustion found in patients suffering from chronic diseases and/or tumours due to changes in e.g., IL-2 production [96].

Other immune responses are also affected by a low-shear environment; inflammation, specifically adaptation of the vasculature (release of vasoactive factors [119]), is determined by the vessel wall state which composes of endothelial cells and mesenchymal stem cells [120]. Microgravity analogues have been shown to exacerbate the effect of endothelial cell activation by inflammatory mediators [121]. However, endothelial cell adhesive cascade molecule expression is not affected by the low-shear environment [121].

### 4.4. Cytokines

Cytokines are vital to the immune system and immunological crosstalk. They are small, secreted proteins influencing communication and interaction between cells [122]. The immune response to a pathogen is affected by the low-shear environment which alters the cytokine profile and consequently the function and proportion of leukocytes [73].

IL-6, which is altered in dendritic cells grown under microgravity, plays an important regulatory role in both the innate and adaptive immune system and is produced after stimulation by the majority of nucleated immune cells and plays an important role in the response to bacterial infection [123]. Studies on interleukin production and associated TLRs (toll-like receptors) during spaceflight have given inconclusive results [123]. Studies on samples retrieved after spaceflight has shown that immune cells expressing TLR2 and TLR4 both increase [124] and decrease in expression [125]. IL-6 is one of many cytokines that have been reported to have altered levels during spaceflight, studies have shown that many more cytokines exhibit altered levels depending on host health. For instance, astronauts suffering from latent virus reactivation show elevated levels of IL-1 alpha, IL-4, IL-6, IL-8, IL-10, IL-12p70, IL-13, interferon-gamma, eotaxin, and IP-10 [126]. This illustrates the changes in cell signalling in the microgravity environment and begins to reveal the scope of cytokine changes in this extreme environment [127]. Differences in adaptive reactions (i.e., changes in cytokine production) with various cytokines help to show how different parts of the immune system adapt to spaceflight. IL-4, IL-6, IL-8, and IL-10 adaptive reactions were found six months after spaceflight whereas IL-2, TNF alpha, and interferon-gamma adaptive reactions were found after only 12 days of spaceflight [128].

## 5. Concluding Remarks

Both bacteria and immune cells can be influenced by growth under a low-shear environment created by microgravity or an analogue. Bacteria exhibit increased proliferation, biofilm formation, and virulence gene expression making them an increased health risk, which when combined with immune dysfunction in microgravity increases the risk of opportunistic infection. The impairment of pathogen recognition and immunological crosstalk impedes and diminishes the immune response from the very early stages of disease progression. Changes in cytokine expression and production in addition to this allow for an increased chance of successful disease progression from initial colonization. 

Furthermore, impairment of immune cell function reduces the ability of the immune system to clear an infection, once more promoting chronic disease progression. Prolonged immune repression upon return to Earth gravity conditions is also a significant health concern.

Immune responses in microgravity are an exciting area of research with many unexplored avenues yet to be investigated, especially the effects of long-term spaceflight. It has highlighted many obstacles that will need to be overcome before long manned missions to other celestial bodies and deep space exploration can occur. 

The most important and compelling areas of research going forward should be how immune cell differentiation is inhibited. Additionally, the immune response to bacterial stimuli needs to be further elaborated upon to discover as to what extent the recognition of bacteria and subsequent signalling and host response is inhibited.

With respect to bacteria, the development of bacterial antibiotic tolerance and biofilms is a major issue that needs to be addressed for long term space flight to be a safer venture.

## Figures and Tables

**Table 1:** Bacterial acceptability limits outlined by NASA [28].

Time Taken	Air	Surface	Water
Preflight	300 CFU m^−3^	500 CFU 100 cm^−2^	50 CFU mL^−1^
Inflight	1000 CFU m^−3^	10,000 CFU 100 cm^−2^	50 CFU mL^−1^

**Table 2:** Summary of bacterial response in spaceflight and microgravity analogue studies.

Name	Low-Shear Environment	Studies	Major Findings
*Mycobacterium marinum*	Rotating Cell Culture System	[31]	562 genes altered transcription level after short growth, 328 after long growth periods. Downregulation of Metabolism. Increases sensitivity to hydrogen peroxide.
*Ralstonia pickettii*	Spaceflight samples in Rotating Cell Culture System	[30]	Increased growth rate
*Escherichia coli*	Rotating Cell Culture System	[32,33,34,35]	Shorter replication time, increased survivability in J774 macrophages, increased resistance to osmotic stress, heat and acid. Increase in biofilm thickness and biomass.
*Salmonella enterica serovar typhimurium*	Rotating Cell Culture System	[36]	Shorter replication time, increased survivability in J774 macrophages, increased resistance to osmotic stress, heat and acid.
*Streptococcus mutans*	Rotating Cell Culture System	[37,38]	153 genes upregulated two-fold or more, 94 genes downregulated two-fold or more
*Lactobacillus acidophilus*	Rotating Cell Culture System	[39]	Shortened lag phase, increased growth rate, increased antibiotic resistance, increased acid and bile resistance.
*Bacillus subtilis*	Spaceflight	[40]	55 genes upregulated (biofilm formation associated genes), 36 genes downregulated (anaerobic respiration associated genes).
*Pseudomonas aeruginosa*	Spaceflight	[41,42,43]	Different biofilm architecture to that formed under Earth gravity.
*Klebsiella pneumoniae*	Rotating Cell Culture System	[44]	Enhanced biofilm formation, thicker biofilms, increased cellulose production.
*Vibrio fischeri*	Rotating Cell Culture System	[45]	Hfq mutant studies.
*Staphylococcus aureus*	Rotating Cell Culture System Spaceflight	[46] [4]	Antibiotic resistance increases. Cell wall changes.

**Table 3:** Response of Archaea to a low-shear environment.

Name	Low-Shear Environment	Studies	Major Findings
*Haloferax mediterranei*	Rotary Cell Culture System	[64]	Increased resistance to bacitracin, rifampicin and erythromycin
*Halococcus dombrowkskii*	Rotary Cell Culture System	[64]	Reduced cell aggregation
*Haloarcula argentinesis RR10*	Rotary Cell Culture System	[65]	Increased production of ribosomal proteins, became multi-drug resistant, evidence of antibiotic efflux pump
